# Variable extent of parallelism in respiratory, circulatory, and neurological traits across lake whitefish species pairs

**DOI:** 10.1002/ece3.469

**Published:** 2013-01-29

**Authors:** Melissa L Evans, Lauren J Chapman, Igor Mitrofanov, Louis Bernatchez

**Affiliations:** 1 Institut de Biologie Intégrative et des Systèmes, Pavillon Charles-Eugène-Marchand1030 Avenue de la Médecine, Université Laval, Québec, Québec, G1V 0A6, Canada; 2 Biology Department, McGill University1205 Avenue Docteur Penfield, Montréal, Québec, H3A 1B1, Canada

**Keywords:** Adaptive radiation, brain, *Coregonus clupeaformis*, ecology, gill, heart ventricle, morphological divergence, speciation

## Abstract

Parallel adaptive radiation events provide a powerful framework for investigations of ecology's contribution to phenotypic diversification. Ecologically driven divergence has been invoked to explain the repeated evolution of sympatric dwarf and normal lake whitefish (*Coregonus clupeaformis*) species in multiple lakes in eastern North America. Nevertheless, links between most putatively adaptive traits and ecological variation remain poorly defined within and among whitefish species pairs. Here, we examine four species pairs for variation in gill, heart, and brain size; three traits predicted to show strong phenotypic responses to ecological divergence. In each of the species pairs, normals exhibited larger body size standardized gills compared to dwarfs – a pattern that is suggestive of a common ecological driver of gill size divergence. Within lakes, the seasonal hypoxia experienced in the benthic environment is a likely factor leading to the requirement for larger gills in normals. Interestingly, the morphological pathways used to achieve larger gills varied between species pairs from Québec and Maine, which may imply subtle non-parallelism in gill size divergence related to differences in genetic background. There was also a non-significant trend toward larger hearts in dwarfs, the more active species of the two, whereas brain size varied exclusively among the lake populations. Taken together, our results suggest that the diversification of whitefish has been driven by parallel and non-parallel ecological conditions across lakes. Furthermore, the phenotypic response to ecological variation may depend on genetic background of each population.

## Introduction

Species arising through adaptive radiation processes represent enigmatic examples of evolution by natural selection (Schluter 2000; e.g., Seehausen 2006). They are also useful models for examining how ecology structures phenotypic diversity given that character displacement is by definition driven by competition for ecological resources (Taylor 1999; Schluter 2000; Day and Young 2004). Some of the best-described examples of adaptive radiations occurring in temperate systems are those of whitefish species in eastern North America and Europe (*Coregonus* spp.; Bernatchez 2004; Bernatchez et al. 2010; Vonlanthen et al. 2012). In the North American lake whitefish (*C. clupeaformis*), a limnetic “dwarf” species has evolved in multiple lakes in the St. John River basin and remains reproductively isolated from the ancestral benthic “normal” species. The sympatric diversification of whitefish was initiated through the secondary contact of two populations that diverged in allopatry during the last glaciation (Schluter and McPhail 1993; Pigeon et al. 1997). Competition for resources following secondary contact ultimately led to the colonization of the limnetic environment by whitefish and the specialization by dwarfs to limnetic ecological conditions.

Contemporary populations of dwarf and normal whitefish occupy different trophic positions within lakes (Bernatchez et al. 2010). Differences in trophic ecology, including predation pressure, prey communities, and associated abiotic conditions may drive complex relationships between phenotype and environment (Landry et al. 2007; Landry and Bernatchez 2010). Indeed, phenotypic and genetic divergence between dwarfs and normals is well documented for traits associated with trophic position (e.g., Lu and Bernatchez 1999; Rogers et al. 2002). For example, dwarfs show a greater number of gill rakers compared to normals, which may facilitate the retention of planktonic prey while filter feeding in the limnetic zone (Lu and Bernatchez 1999; Bernatchez 2004). Also, differences in prey type and the increased predation pressures found in limnetic compared to benthic environments may explain the evolution of more active swimming behaviors in dwarfs compared to normals (Rogers et al. 2002; Kahilainen and Lehtonen 2003; Rogers and Bernatchez 2007). Ecologically driven divergence in other phenotypic traits is likely given that the trophic positions of dwarfs and normals are associated with many differences in the physico-chemical and biotic environment (Landry et al. 2007; Landry and Bernatchez 2010). Furthermore, non-parallel environmental conditions and differences in the genetic backgrounds of whitefish populations may play critical roles in explaining patterns of trait variation (e.g., Evans et al. 2012; Evans and Bernatchez 2012; also see Rosenblum and Harmon 2011; Kaeuffer et al. 2012). Nevertheless, outside of a few well-studied characters such as gill raker number (e.g., Lu and Bernatchez 1999; Siwertsson et al. 2010), the potential for phenotypic trait divergence related to ecological variation remains poorly defined across whitefish populations.

The objective of this study was to expand our examination of the phenotypic characters involved in the ecological divergence of dwarf and normal whitefish. For this, we targeted three organs strongly linked to fitness and the environment: the gills, heart, and brain.

As the primary surface involved in physical and chemical exchange with the external environment, the fish gill is critical to both homeostatic regulation and ability to deal with environmental heterogeneity (Hughes 1966; Wood and Soivio 1991). In addition to scaling negatively allometrically with body mass, gill surface area tends to be larger in more active fish species (see Gray 1954; Palzenberger and Pohla 1992; Bernal et al. 2001). Furthermore, *among fish species*, variation in gill size (gill surface area and/or gill filament length) has been related to dissolved oxygen (DO) availability in the environment (e.g., Galis and Barel 1980; Mazon et al. 1998; Chapman and Hulen 2001). Dissolved oxygen is also a strong predictor of gill size variation *among populations* of fishes. For example, in the African cichlids *P. multicolor* and *Astatoreochromis alluaudi*, the African cyprinid *Barbus neumayeri*, and the New World sailfin molly *Poecilia latipinna*, populations from hypoxic environments are characterized by larger gills than conspecifics from well-oxygenated waters (Chapman et al. 2000, 2008; Timmerman and Chapman 2004; Binning et al. 2010). In whitefish, dwarfs exhibit higher activity levels compared to normals, but normals forage in the benthic zone, which experiences severe seasonal hypoxia in some lakes (Rogers et al. 2002; Landry et al. 2007). Comparisons of the two species within and among lakes should help to tease apart the potential influence of differential activity levels and DO on gill size. If DO is the primary driver of gill size, normal whitefish should exhibit larger gills relative to body size compared to dwarfs, whereas we could expect the reverse pattern if activity levels drive gill size. It is also possible that the two ecological challenges (low DO and high activity) could lead to the requirement for large gills in both dwarf and normal whitefish. However, comparisons of gill size across lakes that experience non-parallel degrees of oxygen depletion should provide insight into gill size response to this ecological factor. Indeed, we take this approach in our study comparing two lakes from Maine (Cliff Lake and Indian Pond) that become strongly hypoxic in the benthic zone in late summer to two lakes in Québec (East Lake and Témiscouata Lake) that show only moderate declines in DO.

The cardiovascular system is also tightly linked to metabolism and the requirement for oxygen delivery (Farrell and Jones 1992). Heart size tends to scale isometrically with body mass in fishes (Farrell and Jones 1992; e.g., Clark and Farrell 2011). However, interspecific variation in cardiac anatomy and size-adjusted heart mass is high, reflecting adaptations to different habitats and activity levels (Gamperl and Farrell 2004). For example, studies of size-matched species with differing activity levels have shown that active species tend to have larger hearts (Weibel et al. 1991). Differences in activity are generally reflected through ventricular mass as larger ventricles should increase blood pressure and cardiac stroke volumes (Farrell and Jones 1992). Furthermore, cardiac function must be sustained during hypoxia exposure, and there is evidence that fish species subjected to hypoxia show morphological and physiological adjustments to enhance their capacity to function under low DO (Gamperl and Farrell 2004; Lague et al. 2012). In whitefish, we predict that differences in activity levels between the species and/or exposure to seasonal hypoxia could drive divergence in heart ventricle size.

In addition to evolutionary history, the ecological conditions experienced by fishes are considered primary factors shaping brain size (Kotrschal et al. 1998). Studies have shown that factors such as habitat complexity, food habits, body form and locomotion, and predation pressures may influence brain morphology and size (Bauchot et al. 1977, 1989; Huber and Rylander 1992; Huber et al. 1997; Kotrschal et al. 1998). Under hypoxia, individuals will face energetic trade-offs between the maintenance of brain tissue and investment into other functions (Chapman and Hulen 2001; Poulson 2001; Safi et al. 2005). Indeed, brain size variation has been associated with intra- and interspecific variation in DO availability in several studies of fishes (e.g., Chapman et al. 2000, 2008; Chapman and Hulen 2001). Given the multifunctionality of the brain and associations between brain mass and ecology, the differing trophic positions inhabited by the dwarf and normal whitefish could lead to profound differences in relative brain size.

Here, we examine how gill size, heart ventricle mass, and brain mass scale with body mass in dwarf and normal whitefish from Cliff Lake and Indian Pond in Maine, USA, and East Lake and Témiscouata Lake in Québec, Canada. Species pairs from Maine generally exhibit higher levels of phenotypic and genetic divergence than those from Québec (Lu and Bernatchez 1999; Campbell and Bernatchez 2004; Renaut et al. 2011). The degree of divergence exhibited between dwarf and normal whitefish is most likely related to the differing postglacial colonization histories of each lake (i.e., genetic background) and differences in ecological conditions found across lakes (Landry et al. 2007). East Lake and Indian Pond were colonized by the Acadian lineage of whitefish only, Témiscouata predominantly by Acadian and to a lesser extent Mississippian lineages, and Cliff Lake by Acadian and Atlantic lineages (Pigeon et al. 1997; Lu et al. 2001). Hence, we test for the effects of lake, species, and their interaction on body size standardized trait size and use the observed patterns of trait divergence to infer the potential role that ecological and genetic differences play in promoting phenotypic diversification.

## Methods

### Sample collection

**Figure 1:**
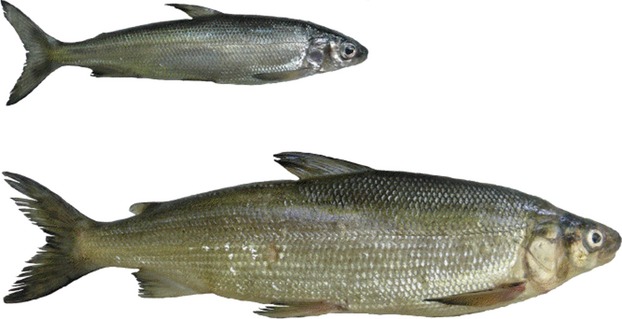
Adult dwarf (upper) and normal (lower) lake whitefish.

Lake whitefish were sampled using gill nets from Cliff Lake and Indian Pond in Maine, USA, in June 2010, and from East and Témiscouata lakes in Québec, Canada in July 2010. All lakes are found within the St. John River watershed (see Landry et al. 2007). For each individual, we determined body mass and length in the field. Subsequently, the whitefish were stored on dry ice for transport to the laboratory. We limited our analyses to sexually mature fish for comparison. The gross phenotypic differences observed between mature dwarf and normal whitefish are shown in Figure 1.

### Gill metrics

We quantified gill size in five dwarf and five normal whitefish from each of the lakes with the exception of Indian Pond, for which we measured gills from five dwarf and four normal whitefish. To characterize gill size in each individual, we removed the four gill arches from the left branchial basket and fixed the arches in 4% paraformaldehyde. On each gill arch, we examined five metrics representing the size and shape of the filamentous part of the gills, that is, the portion of the gill responsible for gas exchange. Characters measured included: total gill filament length (TGFL), average filament length (AFL), total gill filament number (TFN), total area of the filamentous portion of the hemibranch (THA), and total perimeter of the filamentous portion of the hemibranch (THP).

**Figure 2:**
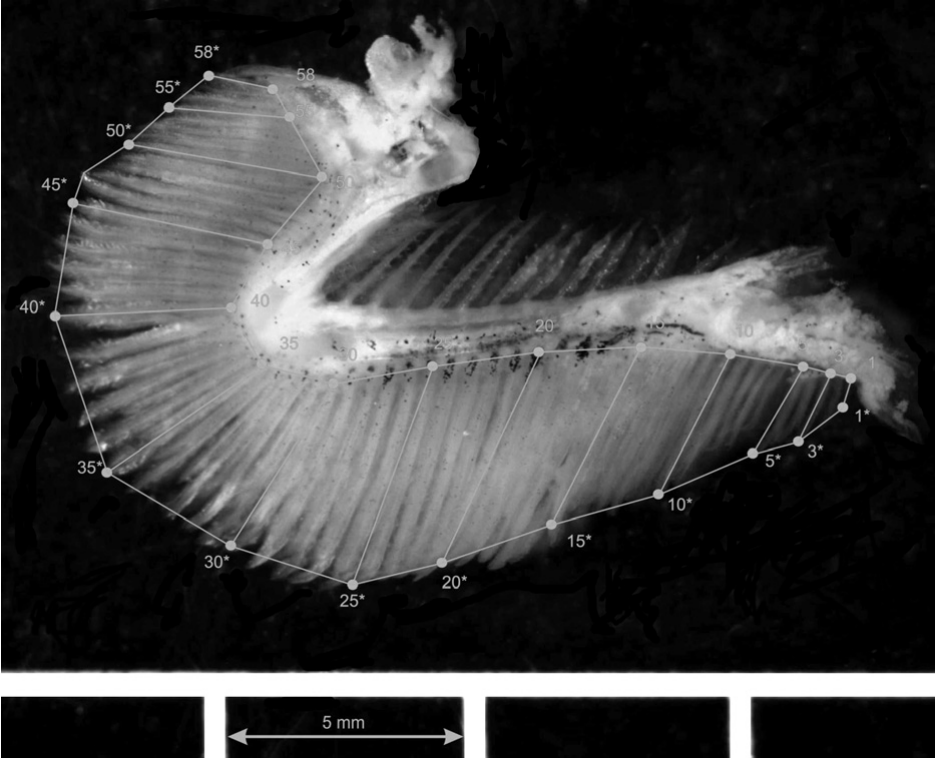
Estimating gill size in lake whitefish. The figure shows, as an example, one of the four gill arches measured per whitefish. For each hemibranch (i.e., each side of the arch), we took a photograph and used the photograph to digitally estimate the length of every fifth filament, the total filament number, the area of the hemibranch (filamentous portion), and the perimeter of the hemibranch (filamentous portion), as depicted.

To quantify TGFL, each gill arch was separated and laid flat on a microscope slide. For each side (hemibranch) of each arch, the length of every fifth gill filament was measured (Fig. 2). Two successive measurements were averaged to estimate the length of the filaments falling between the measured filaments. Filament lengths were summed for the eight sides of the four arches and were multiplied by two to yield an estimate of TGFL. Average filament length (AFL) was calculated as the mean length of all measured filaments. The total number of filaments (TFN) was counted on each hemibranch and multiplied by two to produce an estimate of the number of filaments for the branchial basket. To estimate gill arch area, we digitized the filamentous portion of each hemibranch (Fig. 2). The areas were summed across arches and multiplied by two to produce an estimate of the total hemibranch area (THA). Total hemibranch perimeter (THP) was estimated as the sum of the perimeters of the gill arches multiplied by two.

### Heart and brain mass

We collected and weighed whole brains from each of 11 dwarfs and 11 normals from Cliff Lake, 11 dwarfs and 12 normals from Indian Pond, 12 dwarfs and 12 normals from East Lake, and 12 dwarfs and 12 normals from Témiscouata Lake. Brains were removed, blotted, and weighed (wet weight).

We determined the heart ventricle wet mass (blotted dry) from six dwarfs and five normals from Cliff Lake, three dwarfs and four normals from Indian Pond, five dwarfs and five normals from East Lake, and five dwarfs and six normals from Témiscouata Lake.

### Statistical analyses

Log_10_ transformations were applied to all morphological variables so as to follow a near-normal distribution (Kolmogorov D > 0.05). Linear regression was used to examine the relationships between log_10_-transformed body mass and log_10_-transformed brain and heart mass and gill size. We found no effect of the interactions between species or lake and body mass on the gill size metrics or heart ventricle or brain mass (analysis of covariance: *P* > 0.08), therefore all dwarf and normal whitefish across all lakes were included in our regression analyses. The scaling exponent (*b*) and factor (*a*) for each body mass–organ relationship was determined according to the model of Schmidt-Nielsen (1984), where physiological traits are predicted to scale with body mass (*M*_b_) according to the general equation; *aM*_b_^*b*^.

There is little to no overlap in body size between adult dwarf and normal whitefish in any of the lakes examined, which may confound comparisons of traits that scale allometrically with body size. Thus, in order to compare gill size between the species, we standardized each of the gill size metrics to a common body mass taking into account the allometric relationship between body mass and gill size using the equation: *Y*_std_ = *Y*_obs_(*M*_avg_/*M*_obs_)^β^, where *Y* is the gill metric, *M* is body mass, subscripts std, obs, and avg refer to the body size standardized, observed (actual), and average (for all fish) measures, respectively, and β represents the slope of the relationship between the gill metric and body mass across all lakes and species (Reist 1986; Crispo and Chapman 2010). The β values were obtained from analyses of covariance (ANCOVAs) for each of the five gill metrics (log_10_-transformed) and which included lake and species as fixed factors, lake × species, and log_10_-transformed body mass as a covariate.

The covariate often used for measures of gill size is body mass because of the relationship between metabolic rate and body mass. However, we also standardized each of the gill size estimates to a common body length, because dwarf and normal whitefish differed marginally (ANCOVA for intercepts, *P* = 0.074) in body condition in our samples. Following standardization to common body mass or length, principal components analysis (PCA) of the five standardized gill size metrics was used to describe variation in overall gill size.

Using a common body mass to standardize the gill size metrics, the first two components obtained from our PCA explained 67.1% and 19.8% of the variance in overall gill size, respectively. Principal component one (PC 1) exhibited positive loadings for all five of the metrics (TGFL: 0.927; AFL: 0.872; TFN: 0.421; THA: 0.955; THP: 0.803), indicating that this component is a good estimate of overall gill size. In contrast, principal component two (PC 2) primarily reflected variation in TFN (loading: 0.887) and to a lesser extent TGFL (loading: 0.221). The PCA of gill size standardized by common body length gave similar results to those from the PCA of the body mass standardized traits. Specifically, the first two principle components explained 69.6% and 18.7% of the variance in overall gill size, respectively. Principal component 1 exhibited positive loadings for all five of the gill metrics (TGFL: 0.940; AFL: 0.880; TFN: 0.463; THA: 0.961; THP: 0.826), whereas TFN and TGFL exhibited strong and weak positive loadings, respectively, on the second component (TFN: 0.878; TGFL: 0.164). We used two-way ANOVA to partition variance in the first two principal components to lake, species, and lake × species effects.

We calculated the relative mass of the heart ventricle and brain as a percentage of total wet body mass and used one-way ANOVA to examine how log_10_-transformed relative ventricle or brain mass varied between dwarf and normal whitefish; these models also included population as fixed factors. We then used ANCOVA to partition variation in log_10_-transformed brain and heart ventricle mass to lake, species, and lake×species effects while controlling for differences in body mass (i.e., log_10_-transformed body mass was included in each of the models as a covariate). Tukey's posthoc tests of honestly significant differences (HSD) were used to examine where the specific differences occurred in all models. All statistical analyses were run in JMP version 9.0 (SAS Institute, Cary NC), means are reported ±1 SE, and a threshold significance value, α = 0.05, was used throughout.

## Results

### Allometric scaling of gill, heart ventricle, and brain size

**Figure 3:**
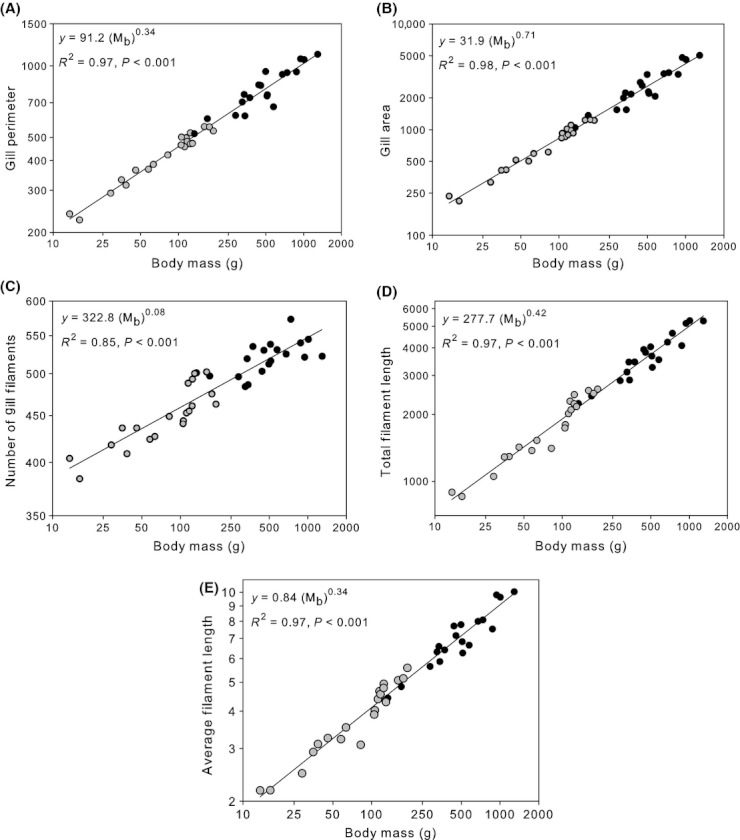
Relationships between body mass and five gill size metrics in the lake whitefish. Points corresponding to dwarf and normal whitefish are shown in gray and black, respectively. The regression line is fitted to all points. The fit of the model is shown in each figure, as is the equation indicating how each of (A) gill perimeter (mm), (B) gill hemibranch area (mm^2^), (C) number of gill filaments, (D) total gill filament length (mm), and (E) average gill filament length (mm) scale with body mass in whitefish. The gill size metrics are plotted against body mass on bilogarithmic scales.

**Figure 4:**
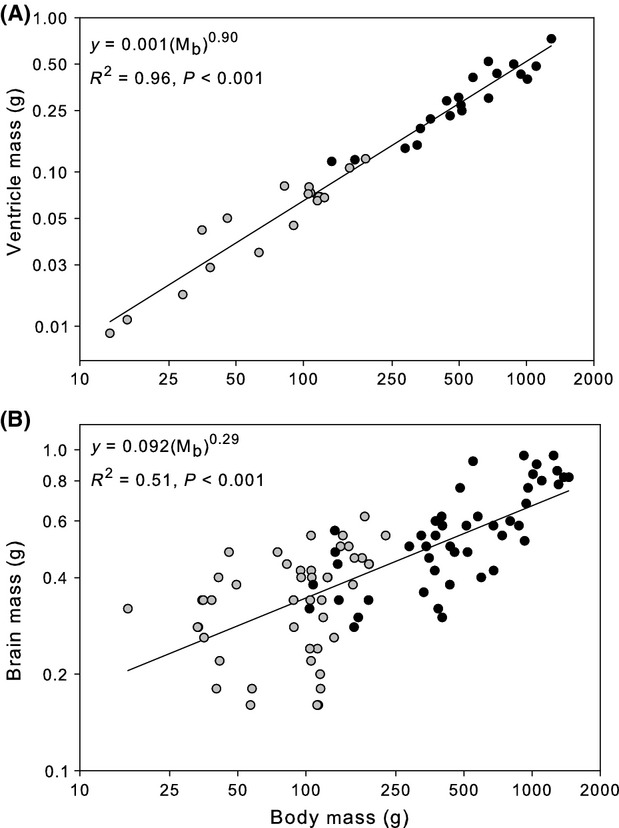
Relationships between body mass and each of heart ventricle mass (A) and brain mass (B) in the lake whitefish. Points corresponding to dwarf and normal whitefish are shown in gray and black, respectively. The regression line is fitted to all points. The fit of the model is shown in each figure, as is the equation indicating how ventricle mass and brain mass scale with body mass in whitefish. Ventricle and brain masses are plotted against body mass on bilogarithmic scales.

Body mass was strongly positively associated with gill size, heart ventricle mass, and brain mass (Figs. 3–4). Each of the gill size metrics exhibited negative allometric relationships with body mass, although their scaling exponents varied (Fig. 3). Ventricle mass scaled nearly isometrically to body mass (Fig. 4A). Brain mass was also positively associated with body mass, though to a lesser extent than ventricle mass, and with a scaling exponent of 0.29, indicating that as body size increases, relative brain mass decreases (Fig. 4B).

### Lake and species effects on gill size variation

**Figure 5:**
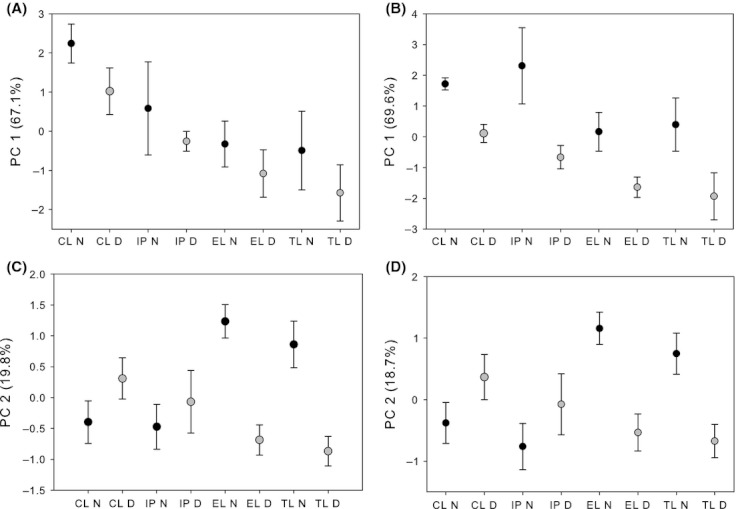
Variation in body mass or length standardized principal components (PC) of gill size in dwarf (D-gray circles) and normal (N-black circles) lake whitefish from Cliff Lake (CL) and Indian Pond (IP) in Maine, USA, and East Lake (EL), and Témiscouata Lake (TL) from Québec, Canada. Principal component 1 (PC1) exhibited positive loadings for all five gill size metrics (TGFL, AFL, TFN, THA, THP) when standardized by body mass (A) or length (B), whereas TFN is the primary metric explaining variation in principal component (PC 2) for both body mass (C) and body length (D) standardized metrics. Mean component scores are reported ±1 SE.

Two-way ANOVA revealed that both lake and species were associated with variation in gill PC 1. Specifically, whitefish from Cliff Lake and Indian Pond exhibited significantly larger gill PC 1 scores than East and Témiscouata whitefish (Table 1, Fig. 5A–B). Lake was a strong predictor of gill PC 1 regardless of whether we used body mass or length standardized gill metrics in our PCA. In each of the lakes examined, normal whitefish exhibited larger gill PC 1 scores than dwarf whitefish (Table 1, Fig. 5). The trend towards larger gills in normals was significant for the length standardized gill metrics, but only nearly significant for the body mass standardized metrics.

**Table 1:** Analysis of variance of the first and second principal components of gill size in lake whitefish from four lakes in Maine, USA, and Québec, Canada

	PC 1 Mass standardized			PC 2 Mass standardized			PC 1 Length standardized			PC 2 Length standardized		
				
	F	DF	*P*-value	F	DF	*P*-value	F	DF	*P*-value	F	DF	*P*-value
Model	3.05	7,31	**0.015**	4.82	7,31	<**0.001**	5.21	7,31	**<0.001**	4.05	7,31	**0.003**
Lake	5.81	3	**0.003**	0.81	3	0.494	4.36	3	**0.011**	1.42	3	0.255
Species	3.79	1	0.061	6.71	1	**0.014**	23.51	1	**<0.001**	2.87	1	0.100
Lake×Species	0.05	3	0.986	8.04	3	<**0.001**	0.44	3	0.724	7.07	3	<**0.001**

The contribution of lake, species (dwarf, normal), and the interaction between lake and species were examined for both body mass and length standardized principal components of gill size. As outlined in the methods, principal component 1 (PC 1) exhibited positive loadings for all five gill metrics, whereas number of gill filaments was the primary factor influencing component two (PC 2). Significant *P*-values (*P* < 0.05) are indicated in bold.

For both body mass and body length standardized inputs, the interaction between lake and species was strongly associated with PC 2 of gill size, which largely reflected variation in total filament number. Tukey's *HSD* tests revealed that East and Témiscouata normal whitefish exhibited higher PC 2 scores than their dwarf counterparts (*P* < 0.05; Fig. 5C–D). In contrast, dwarf and normal whitefish from Cliff Lake and Indian Pond did not differ significantly in PC 2 scores, albeit there was a trend for dwarfs to show higher scores than normals (Fig. 5C–D).

### Lake and species effects on heart and brain size variation

**Figure 6:**
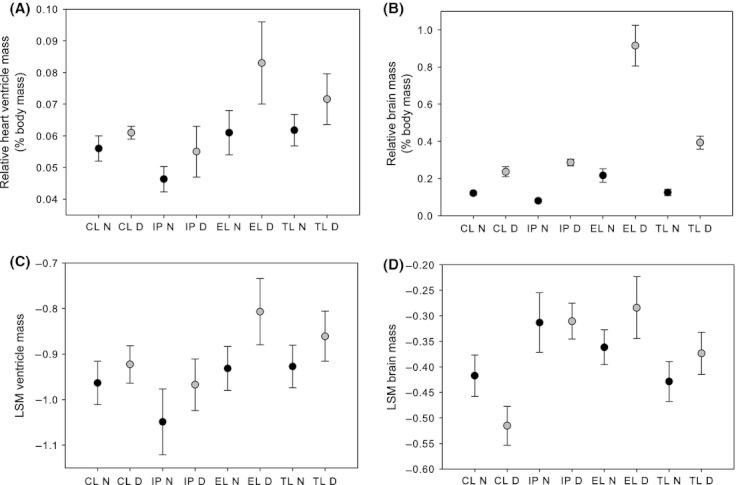
Variation in heart ventricle and brain mass in dwarf (D-gray points) and normal (N-black points) lake whitefish from Cliff Lake (CL) and Indian Pond (IP) in Maine, USA, and East Lake (EL), and Témiscouata Lake (TL) from Québec, Canada. Mean relative ventricle (A) and brain (B) masses are shown as a percent of body mass ± 1 SE. Also plotted are least squares means (LSM) of ventricle (C) and brain (D) masses ± 1 SE in the species in each of the four lakes. LSM scores were derived from ANCOVA examining the contribution of lake, species, their interactions, and body mass to variation in ventricle and brain mass.

One-way ANOVA revealed that both relative ventricle mass (ventricle mass as a percentage of body mass) and relative brain mass were significantly larger in dwarfs compared to normals (ventricle mass: F_4,34_ = 4.58, *P* = 0.005; brain mass; F_4,88_ = 65.03, *P* < 0.001; Fig. 6A–B). We also used ANCOVA to examine lake and species effects on body mass standardized brain and heart ventricle masses. Neither lake nor species were significant predictors of ventricle mass (Table 2), albeit there was a non-significant trend toward larger ventricle masses in dwarfs compared to normals (Fig. 6C). ANCOVA also revealed significant differences among lakes in brain mass, with East Lake and Indian Pond whitefish showing significantly larger brains than whitefish from Cliff Lake (Tukey's *HSD*: *P* < 0.05; Fig. 6D). Species was not associated with brain mass; however, we did observe a near significant lake × species effect on brain mass (Table 2). Specifically, East and Témiscouata dwarfs showed a trend toward larger brain masses compared to normals, whereas Cliff Lake dwarfs showed smaller brain masses than normals (Fig. 6D).

**Table 2:** Results from analysis of covariance models examining the contribution of species (dwarf, normal) and lake to variation in heart ventricle and brain mass in lake whitefish (*Coregonus clupeaformis*)

	Heart ventricle			Brain		
		
	F	DF	*P-*value	F	DF	*P-*value
Model	122.39	8,30	**<0.001**	20.15	8,84	<**0.001**
Body Mass	182.61	1	**<0.001**	20.00	1	<**0.001**
Lake	2.17	3	0.113	8.99	3	<**0.001**
Species	1.21	1	0.281	0.03	1	0.864
Lake×Species	0.28	3	0.836	2.44	3	0.069

Body mass was included as the covariate in each model, and the interactions between lake and species were also examined. Significant *P*-values (*P* < 0.05) are indicated in bold.

## Discussion

To increase our understanding of the traits involved in whitefish species diversification, we quantified inter- and intra lake variation in three organs strongly linked to fitness and ecological variation: the gills, heart, and brain. Evaluating the potential influence of divergent ecologies on whitefish character variation is complicated by the body size differences found in dwarfs and normals. Indeed, our results show a clear influence of body size on gill size, heart ventricle mass, and brain mass in whitefish. However, once body size differences are taken into account, the observed patterns of size variation in each of the traits indicate that ecological differences may be driving some combination of genetic variation and plasticity in these components of phenotype.

### Gill character divergence

The ability of an organism to take-up oxygen from the environment is largely dependent on structural parameters associated with the lungs or gills (Weibel et al. 1991). Our results indicate that gill size has diverged significantly between dwarf and normal lake whitefish, with normals exhibiting larger gills compared with dwarfs in each of the four lakes examined. Although the size of respiratory structures may increase in response to higher metabolic oxygen demands (Weibel et al. 1991; Maina 2002; Nilsson 2007), our results suggest that this is not the primary factor driving the observed differences in gill size between dwarf and normal whitefish, as dwarfs exhibit the higher metabolic rate of the two (Trudel et al. 2001). Other studies have shown that fishes may alter gill size in response to low oxygen (e.g., Chapman et al. 2000; Sollid et al. 2003; Nilsson 2007; Crispo and Chapman 2010), and we suggest that exposure to low levels of dissolved oxygen is a likely factor influencing gill size in whitefish. Seasonal declines in oxygen saturation occur toward the end of summer in each of the lakes examined in this study, resulting in severe hypolimnetic hypoxia in Cliff Lake and Indian Pond and more moderate declines in Témiscouata Lake and East Lake (Landry et al. 2007). Thus, for the normal whitefish, larger gills may facilitate hypoxia tolerance as they forage in the benthos (see Chapman and Hulen 2001). Gill size variation between the whitefish species and among lakes could be related to phenotypic plasticity and/or an evolutionary response to seasonal hypoxia. In the cichlid *P. multicolor*, fish reared under normoxia and hypoxia show developmental plasticity in gill filament length and gill surface area (Chapman et al. 2000, 2008). Reversible plasticity in gill size (non-developmental) has been observed in crucian carp (*Carassius carassius*) and goldfish (*C. auratus)* (Sollid and Nilsson 2006). If variation in whitefish gill size is a reversible plastic response to seasonal hypoxia, we would not have expected to see a strong difference between species or among lakes, as we sampled fish prior to the onset of the hypolimnetic oxygen depletion that occurs in late summer (Landry et al. 2007). Thus, it is likely that the observed divergence in gill size is related to developmental phenotypic plasticity or genetic differences between dwarf and normal whitefish.

Spatial trade-offs between gill structures associated with trophic position and respiration may also influence gill morphology in fishes (Chapman et al. 2000, 2008; Binning et al. 2010). Dwarf whitefish show a larger number of gill rakers compared to normal whitefish in three of the populations examined in this study; Cliff Lake, Indian Pond, and Témiscouata Lake, and which is associated with the zooplankton-based foraging ecology of dwarfs (Lu and Bernatchez 1999; Landry et al. 2007). It is possible that a larger gill raker number could constrain the size of the filamentous portion of the gill in dwarfs. However, we consider spatial trade-offs an unlikely driver of gill respiratory morphology in whitefish for two reasons: first, dwarf and normal whitefish in these lakes differ by only 1–3 gill rakers on average (out of ∼25), and second, even in East Lake, where dwarfs and normals do not differ in gill raker number (Lu and Bernatchez 1999), the divergence in gill size remains evident. Taken together, we suggest that seasonal exposure to hypoxic conditions is the most likely ecological driver of gill size variation between normal and dwarf whitefish.

Two limitations of our study must be considered in evaluating the strength of our inferences. First, size differences between mature dwarf and normal whitefish complicate comparisons of traits that scale with body size, as there is little overlap in the size range of mature individuals of the two species. The effect of body size on oxygen uptake capacity in fishes has been a subject of debate for decades. In their recent review of scaling of hypoxia tolerance in fishes, Nilsson and Ostlund-Nilsson (2008) have argued that the scaling exponent for the relationship between respiratory surface area and body mass is similar to the scaling exponent for metabolic rate and body mass. Therefore, gill surface area should match oxygen uptake requirements over a large body size range and differences in size-adjusted respiratory surface area are more likely to reflect natural selection for increased oxygen uptake capacity rather than physiological scaling. Second, we could not measure the surface area of the gill lamellae because the required method of preservation in the field (samples were deep-frozen for genomic work) could affect the integrity of fragile soft tissues. However, in previous studies of cichlid and poeciliid fish species, populations with larger total filament areas were characterized by a larger gill surface area (*Poecilia latipinna*, Timmerman and Chapman 2004; *Pseudocrenilabrus multicolor,* Chapman et al. 2000, 2008; Crispo and Chapman 2010) and thus our estimates of variation in gill size should be strong predictors of variation in gill respiratory surface area.

In addition to observing differences in gill size between the two species, we observed larger gills in whitefish from the two lakes in Maine, Cliff Lake and Indian Pond, when compared to whitefish from the two lakes in Québec. This result may point to the degree of seasonal hypoxia experienced across lakes as an important driver of gill size, as hypolimnetic oxygen saturation is lower in Cliff Lake and Indian Pond than in East Lake and Témiscouata Lake (Landry et al. 2007). Furthermore, the structural characteristics used by whitefish to achieve larger gills appear to vary between the Maine and Québec lakes. Specifically, normal whitefish from East and Témiscouata lakes showed a larger number of filaments, but smaller gill size overall, compared to whitefish from the lakes in Maine. Because whitefish from Maine and Québec are largely derived from differing ancestral populations (Lu et al. 2001), the morphological pathways used by normals to achieve larger gills in each lake could reflect differing genetic capacities to respond to hypoxia. Studies involving parental forms and hybrids between Maine and Québec whitefish will be necessary to investigate this putative genetic effect on gill structural responses to environmental heterogeneity across lakes.

### Heart ventricle mass

Heart mass tends to scale in direct proportion to body mass in most fishes, although this relationship can vary among populations and species (Farrell and Jones 1992; Clark and Farrell 2011). In whitefish, the shape of the relationship between body mass and heart ventricle mass was close to, but not entirely, isometric, that is, the scaling exponent of < 1 indicates that larger whitefish (i.e., normals) exhibit relatively smaller hearts than smaller whitefish. After controlling for body size, there remained a trend, albeit non-significant, toward larger ventricle sizes in dwarf compared to normal whitefish. Larger heart ventricles in fishes are typically associated with more active swimming behaviors (Farrell and Jones 1992). Indeed, previous studies of whitefish held under common garden conditions have shown that dwarf whitefish are more active swimmers than normal whitefish (Rogers et al. 2002; Rogers and Bernatchez 2007). Little is known about the relative contribution of genetic versus phenotypically plastic changes to ventricle size in fishes, but in mammals, approximately 40% of the variation in heart size appears to be heritable (e.g., Garland et al. 1990; Mahaney et al. 1993). Thus, it is possible that the variation we have observed in ventricle size between dwarfs and normals could derive from plastic or evolutionary responses to activity energetic requirements.

### Brain mass

Whitefish brain mass showed a low body mass scaling coefficient (0.29), suggesting that ecological or evolutionary factors are important drivers of brain mass (Kotrschal et al. 1998). After controlling for body mass-associated effects, we observed significant variation among lakes in brain mass, particularly in dwarfs. Furthermore, dwarfs from East Lake and Témiscouata Lake tended to show larger brains than normals from the same lakes, albeit these differences were not significant. The trend toward smaller brains in normal whitefish could be associated with the energetic constraints imposed by seasonal hypoxia (Chapman and Hulen 2001; Crispo and Chapman 2010). However, we did not observe differences in brain mass between dwarfs and normals in the Maine lakes, where hypoxic conditions are most severe, suggesting that DO levels are not limiting brain size in whitefish.

Dwarf and normal whitefish may also face differences in predation pressure and prey availability and type within and across lakes. Indeed, dwarf European whitefish (*C. lavaretus*) face a greater risk of predation due to their smaller size and open water habitat use compared to normals (Kahilainen and Lehtonen 2003). Bauchot et al. (1989) quantified variation in brain size in 737 species of tropical teleost fishes and found that species that passively avoided predation (e.g., via crypsis) were characterized by a low encephalization index compared to species that used active means of escape. We do not currently have estimates of predation pressure in each of the lakes examined in this study, but it is also possible that predator communities vary among lakes and place differing developmental or selection pressures on brain size (e.g., Gonda et al.^2009^). Among the lakes examined in our study, clear differences also occur in prey community composition and density, which could alter the energetic landscape faced by whitefish across lakes and contribute to interpopulation variation in brain size (Landry et al. 2007; Landry and Bernatchez 2010). A recent study of Catarrhine primates showed that greater seasonal variation in diet was associated with smaller brains, potentially due to the trade-offs associated with maintaining energetically expensive brain tissues when resources are scarce (Van Woerden et al. 2012). Studies of resource fluctuations in each of the whitefish lakes are needed to determine whether availability of prey is a potential factor driving variation in brain size. In the future, detailed examination of the different regions of the brain and prey and predator communities should help to elucidate the potential for changes in brain size in response to ecological pressures.

### Conclusions

Together, our results demonstrate parallel and non-parallel patterns of diversification of the respiratory, cardiovascular, and neurological systems across sympatric whitefish species pairs. The replicated divergence of gill size between dwarfs and normals implicates common ecological drivers of phenotypic diversification across whitefish populations. However, we also observed subtle non-parallelism in the degree of gill size divergence exhibited between species and the mechanisms used to obtain larger gills across populations. The observed patterns of gill size diversification generally match observations of parallelism and non-parallelism at the genetic level (e.g., Lu and Bernatchez 1999; Campbell and Bernatchez 2004; Renaut et al. 2011), suggesting that the ecological speciation of whitefish is complex and underlain by differing genetic and phenotypic pathways. Under a strong phylogenetic framework such as is found in whitefish and other postglacial fishes, studies of ecologically relevant trait variation can be harnessed to reveal how the shared and diverging ecologies faced by organisms shape patterns of diversity.
